# Quality of life, psychological adjustment, and adaptive functioning of patients with intoxication-type inborn errors of metabolism – a systematic review

**DOI:** 10.1186/s13023-014-0159-8

**Published:** 2014-10-25

**Authors:** Nina A Zeltner, Martina Huemer, Matthias R Baumgartner, Markus A Landolt

**Affiliations:** Division of Metabolism, Children’s Research Center and University Children’s Hospital Zurich, Zurich, Switzerland; Department of Psychosomatics and Psychiatry, Children’s Research Center and University Children’s Hospital Zurich, Zurich, Switzerland; Radiz – Rare Disease Initiative Zurich, Clinical Research Priority Program for Rare Diseases, University of Zurich, Zurich, Switzerland; Zurich Center for Integrative Human Physiology, University of Zurich, Zurich, Switzerland; Department of Child and Adolescent Health Psychology, Institute of Psychology, University of Zurich, Zurich, Switzerland

**Keywords:** Health-related quality of life, Psychological adjustment, Adaptive functioning, Inborn errors of metabolism, Inherited metabolic diseases, Organic acidurias, Urea cycle disorders, Maple syrup urine disease, Tyrosinemia

## Abstract

**Background:**

In recent decades, considerable progress in diagnosis and treatment of patients with intoxication-type inborn errors of metabolism (IT-IEM) such as urea cycle disorders (UCD), organic acidurias (OA), maple syrup urine disease (MSUD), or tyrosinemia type 1 (TYR 1) has resulted in a growing group of long-term survivors. However, IT-IEM still require intense patient and caregiver effort in terms of strict dietetic and pharmacological treatment, and the threat of metabolic crises is always present. Furthermore, crises can affect the central nervous system (CNS), leading to cognitive, behavioural and psychiatric sequelae. Consequently, the well-being of the patients warrants consideration from both a medical and a psychosocial viewpoint by assessing health-related quality of life (HrQoL), psychological adjustment, and adaptive functioning. To date, an overview of findings on these topics for IT-IEM is lacking. We therefore aimed to systematically review the research on HrQoL, psychological adjustment, and adaptive functioning in patients with IT-IEM.

**Methods:**

Relevant databases were searched with predefined keywords. Study selection was conducted in two steps based on predefined criteria. Two independent reviewers completed the selection and data extraction.

**Results:**

Eleven articles met the inclusion criteria. Studies were of varying methodological quality and used different assessment measures. Findings on HrQoL were inconsistent, with some showing lower and others showing higher or equal HrQoL for IT-IEM patients compared to norms. Findings on psychological adjustment and adaptive functioning were more consistent, showing mostly either no difference or worse adjustment of IT-IEM patients compared to norms. Single medical risk factors for HrQoL, psychological adjustment, or adaptive functioning have been addressed, while psychosocial risk factors have not been addressed.

**Conclusion:**

Data on HrQoL, psychological adjustment, and adaptive functioning for IT-IEM are sparse. Studies are inconsistent in their methodological approaches, assessment instruments and norm populations. A disease-specific standard assessment procedure for HrQoL is not available. Psychosocial risk factors for HrQoL, psychological adjustment, or adaptive functioning have not been investigated. Considering psychosocial variables and their corresponding risk factors for IT-IEM would allow evaluation of outcomes and treatments as well as the planning of effective social and psychological interventions to enhance the patients’ HrQoL.

## Introduction

Intoxication-type inborn errors of metabolism (IT-IEM) are a group of inborn errors of metabolism (IEM) which share distinct clinical features. The group encompasses urea cycle disorders (UCD), organic acidurias (OA), tyrosinemia type 1 (TYR 1), and maple syrup urine disease (MSUD). The estimated incidence is between 1:8,000 to 1:44,000 for UCD [1,2], about 1:21,000 for OA [2], about 1:100,000 for TYR 1 [3] and about 1:185,000 for MSUD [4].

Patients with IT-IEM share two main clinical features: they have to follow a strict diet, and they live with the permanent risk of metabolic crises. These can be triggered by alterations in diet, common infections, or stress but may also occur without predictive circumstances. In cases of metabolic crises, patients immediately require intensified home care or hospitalisation. Despite such efforts, crises remain life-threatening and may cause organ and central nervous system (CNS) damage [5]. To ensure correct diet and an appropriate reaction in risk situations, patients and their families are obliged to develop extensive knowledge about the disease.

Intense biomedical research in the field of IT-IEM has resulted in substantial advances in treatment and a growing group of surviving patients [6]. These individuals have to cope with stressors such as strict diet, medication, crises management, and uncertainties about the future course of their disease and its consequences. Furthermore patients may have behavioural, cognitive, or psychiatric problems due to the CNS alterations caused by the disease [5], which again impair their psychological functioning [7,8]. As a result, even though treatments have improved, IT-IEM affect the daily life and well-being of patients and their caregivers considerably [9]. Therefore, complementary research from a psychosocial perspective is especially needed [9,10].

Health-related quality of life (HrQoL), psychological adjustment, and adaptive functioning are well-established constructs to describe psychosocial consequences of chronic diseases. HrQoL has been defined as “a patient’s perception of the impact of disease and treatment on functioning in a variety of dimensions, including physical, psychological and social domains” [11], p.126. Adjustment describes the healthy rebalancing of patients to a new condition [12]. We use the more specific term psychological adjustment to refer specifically to emotional, behavioural or social adjustment to a disease. Finally, adaptive functioning is a related term describing “the performance of daily activities required for personal and social sufficiency” ([13], p.6), consisting of the three domains conceptual, practical and social functioning [14]. To date, no consensus has arisen about how HrQoL, psychological adjustment, and adaptive functioning are affected in IT-IEM patients and what factors can influence the well-being of the patients. An overview of findings is lacking. For this reason, we decided to systematically review the current research on HrQoL, psychological adjustment, and adaptive functioning in IT-IEM patients. Our purpose was to answer two research questions:

1. What is the current state of knowledge about self- and proxy-reported HrQoL, psychological adjustment, and adaptive functioning in IT-IEM patients?
2. What are the medical and psychosocial risk factors for HrQoL, psychological adjustment, and adaptive functioning in IT-IEM patients?

## Methods

### Data sources and search strategies

To identify eligible studies for our review, we searched relevant databases with pre-defined search terms. The search was conducted using the following electronic bibliographic databases up to 30 April 2013: *Pubmed*, *Embase*, *Cinahl*, *PsycINFO, Psyndex* and the *Cochrane Database of Clinical Trials and Systematic Reviews. NDLTD (Networked Digital Library of Theses and Dissertations)* and *dissonline.de* were searched to find eligible dissertations. We applied two groups of search terms. Firstly, we employed various disease names referring to IEM and IT-IEM. Secondly, we referred to HrQoL, psychological adjustment, and adaptive functioning by employing these terms: quality of life, life satisfaction, well being, well-being, wellbeing, adjustment, adaption, adaptation, adaptive, psycholog*, psychosocial, psychiatr*, social, emotional, mental health, mental disorder, mental disease, behavior*, behaviour*. To augment the specificity of the search, the IEM/IT-IEM group and the HrQoL/psychological adjustment/adaptive functioning group were connected to each other by the Boolean operator “AND”, whereas terms within the groups were connected by the Boolean operator “OR”. We also took advantage of other options to refine the search when the databases offered them; accordingly, search terms concerning IEM, HrQoL, psychological adjustment, and adaptive functioning were limited to titles and abstracts, and words with multiple possible endings or spellings were completed by wildcards. An additional search for studies was conducted in two ways. First, to minimise publication bias, experts in the field were contacted via e-mail and asked if they were aware of any relevant articles or unpublished data. In addition, the references of relevant articles were screened.

### Study selection

To find eligible studies, we rated all the articles and dissertations found in our systematic search according to pre-defined inclusion and exclusion criteria. Studies were included if the number of participants was N >1 and if the sample contained at least 50% IT-IEM patients or if the results of IT-IEM patients were reported separately. Outcomes had to include a self-, proxy-, or examiner’s report of patients’ HrQoL, patients’ psychological adjustment (psychological, social, behavioural, or emotional adjustment), or adaptive functioning. The assessment of these outcomes had to be completed in a standardised way and reported quantitatively. Reporting of methods and results had to be sufficient for replicability. Finally, reports were accepted if they were written in English, German, French, or Spanish. Articles not fulfilling these criteria were excluded. The selection process was conducted in two major steps. First, one reviewer (N.A.Z.) examined all titles and abstracts. Second, studies that could not be excluded in the first examination were rated in their full-text version by two reviewers (N.A.Z. and M.H.) independently. Inter-rater reliability was substantial [10], with Cohen’s kappa = 0.79. Any disagreements were arbitrated through discussion. The remaining articles were included for data extraction.

### Data extraction and analysis

Two reviewers (N.A.Z. and M.A.L.) extracted the data of the articles independently. Inter-rater reliability was almost perfect [15], with Cohen’s kappa =0.98. Disagreements were resolved through discussion. The great variation between studies regarding design, such as measures and reporting of results, did not permit statistical pooling of data from the individual studies, so meta-analytic calculations were not possible. Instead, effect sizes were calculated whenever possible to attain some comparability between the results. Standardised mean differences, including 95% confidence intervals (CI), were calculated for continuous outcomes using Cohen’s d effect sizes, corrected for small sample sizes [16]. An effect size was considered to be significant if its 95% CI did not include 0, thus considering a significance level of p < .05. According to Cohen’s categories, an effect size is small if d = 0.2 - 0.5, medium if d = 0.5 - 0.8 and large if d >0.8 [17]. Calculations were conducted such that a positive Cohen’s d stands for a higher scoring of the IT-IEM group than of norms. Higher scorings are favourable for all scales, except for two cases: the Child Behaviour Checklist (CBCL) and the Behaviour Assessment System for Children (BASC) (all subscales but adaptive skills) report problem behaviour. Consequently, higher scores signify more problems and are unfavourable outcomes. For dichotomous outcomes, we used Chi-square tests, indicating the strength of the association by Cramer’s V, with p < .05 considered significant. IBM SPSS Statistics for Windows, Version 20.0 was used for all calculations.

## Results

**Figure 1:**
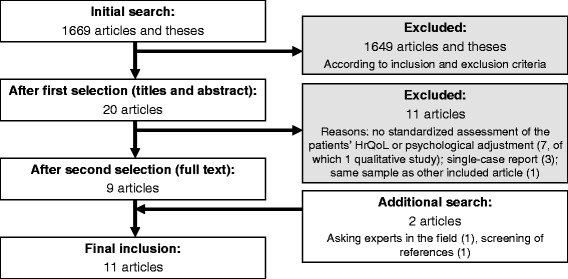
**Flowchart of study selection.**

The initial search of databases revealed 1669 articles and dissertations. After the first selection, 20 articles remained. During the second selection process, we had to exclude another nine articles; three were single-case reports, one had the same sample as another article included, and seven articles lacked standardised assessments of the patients’ HrQoL, psychological adjustment, or adaptive functioning. One of these articles was a qualitative study [18]. One additional, recently published article [19] was found by contacting experts in the field, and another was traced [20] by screening the references of relevant studies. The search and selection process is depicted in Figure 1. Finally, 11 articles remained for further analyses.

### Study description

The main characteristics and results of the articles included in our review are summarised in Table 1. Further information about the assessment instrument used in the studies can be found in Table 2. Detailed analyses of outcome parameters are shown in Tables 3 and 4. All 11 articles were published between 2006 and 2013, seven of them in 2012 or 2013. Four articles have their origins in the United States, two in Germany, and one each in Australia, Belgium, Italy, Poland, and Turkey. All articles are written in English. All of the four disease groups we searched for (UCD, OA, MSUD, TYR 1) are represented in the final selection of studies. Patients diagnosed with MSUD (n = 124) represent the largest group, followed by OA (n = 107), UCD (n = 100) and TYR 1 (n = 8). Seven studies reported outcomes for only one of these disease groups, four studies included patients of multiple groups. From six articles, only subgroup data were extracted. In four studies, we selected IT-IEM patients from the original samples, which integrated patients with IT-IEM and patients with other diseases [19,21–23]. One study reported on IT-IEM patients before and after transplantation. Only the subgroup before transplantation was selected for further analysis [24]. In another study, outcome regarding adaptive functioning was only available for a subgroup [20]. The final sizes considered ranged from N = 4 to N = 92 patients.

**Table 1:** **Main characteristics of the reviewed studies**

**Author, year** ***(origin)***	**Metabolic disease**	**N** ^*^	**Reviewed sample vs. originally reported sample**	**Mean age in years (range)**	**Group of comparison**	**Assessment instrument** ***(report)***	Selected results** (IT-IEM related to group of comparison)
Beauchamp et al., 2009 *(Australia)* [25]	GA I	4	Same	5.8 (5 to 7)	Population norms	• CBCL *(proxy-mother)*	• **Psychological adjustment (CBCL):** No sign. group difference, except for CBCL total scale, where IT-IEM patients show less behavioural problems than the norm population (doubt about reliability of this result)
						• ABAS *(proxy-mother)*	**• Adaptive functioning (ABAS):** No sign. group difference
Cazzorla et al., 2012 *(Italy)* [22]	OTCD, HHH Syndrome, ASA, GA I, MMA, MSUD	15	*Reviewed sample:* only IT-IEM *Orig. sample:* IT-IEM mixed with other diseases (N = 82)	25.6 (17 to 44)	Population norms, other IEM-groups: PKU, Morbus Fabry, pharmacological treatment	• WHOQOL-100 *(self)*	**• QoL (WHOQOL-100):** Compared to population norms: sign. higher QoL for physical domain, lower for environmental domain, no sign. group difference for all other domains
							**• QoL (WHOQOL-100):** Compared to other IEM: no sign. group difference compared to PKU for all domains, sign. higher compared to Morbus Fabry and pharmacologically treated patients in most domains (no sign. group difference for social and environmental domains)
Eminoglu et al., 2013 *(Turkey)* [23]	MA, PA, MSUD *(group includes n = 3 patients with a disease not being an IT-IEM)*	14	*Reviewed sample:* separately reported subgroup, mainly IT-IEM, 3 other IEM *Orig. sample:* IT-IEM mixed with other IEM (N = 68)	4.7 (n.a., SD = 4.3)	Population norms, other IEM-groups: CMD and AMD	• Questionnaire constructed by authors: QoL Scale for Metabolic Diseases *(proxy-parent)*	**• HrQoL (QoL Scale for Metabolic Diseases):** Sign. lower compared to CMD and AMD for school status and health perception domains, sign. lower in physical function domain compared to AMD, similar for other domains
						• Kiddy-, Kid- Kiddo-KINDL *(proxy-parent, self if > = 4 years)*	**• HrQoL (KINDL):** No sign. group difference compared to CMD and AMD for emotional wellbeing domain
Gramer et al., 2013 *(Germany)* [19]	ASLD, GA I, IVA, PA, MSUD	34	*Reviewed sample:* only IT-IEM *Orig. sample:* IT-IEM mixed with other IEM (N = 187)	4 (1.2 to 9.7)	None	Questionnaire constructed by authors, assessing*:*	
						• Perceived burden for the child *(proxy-parent)*	**• Psychological adjustment (Perceived burden for the child):** Rated as low for the majority (50%)
						• Social behavior *(proxy-parent)*	**• Psychological adjustment (Social behaviour):** Rated average for the majority (82%)
Grünert et al., 2013 *(Germany)* [26]	PA	48	Same	5 (5 days to 19)	Population norms	• Kid-KINDL *(self):* n = 18	**• HrQoL (KINDL):** Sign. lower HrQoL for psychological and friends domain, sign. higher for school domain, no group difference for other domains
				• For Kid-Kindl: 11 (5 to 18)		• SDQ *(proxy-parent)*: n = 48	**• Psychological adjustment (SDQ):** More problems in all scales except conduct problems
				• For SDQ: 4 (1 to 18)		n according to age or degree of mental retardation	
Krivitzky et al., 2009 *(USA)* [27]	UCD	92	Same	7.2 (0.4 to 16.75)	Population norms	• ABAS (proxy-parent): all ages	**• Adaptive functioning (ABAS):** General score was sign. lower for all IT-IEM groups (neonatal onset, late onset, patients with/without hyperammonemic history) in the age group of 3-16 years
							**• Adaptive functioning (ABAS):** General score was sign. lower for the IT-IEM patients with a hyperammonemic history, not for the other subgroups, in the age group of < 3 years.
						• CBCL (proxy-parent): for ages 3-16	**• Psychological adjustment (CBCL):** No sign. group difference in internalising and externalising problems
Mazariegos et al., 2012 *(USA)* [20]	MSUD	31	*Reviewed sample:* Patients with results for adaptive functioning *Orig. sample:* Patients with and without results for adaptive functioning (N = 35)	9.9 (1.7 to 32.1) (for N = 35)	Population norms	• ABAS *(self)* or Vineland *(self)* (for this review: only pre-transplantation assessment)	**• Adaptive functioning (ABAS or Vineland):** Sign. lower score for adaptive functioning
							**• Risk factor assessment:** Sign positive correlation between IQ and adaptive functioning
							**• Risk factor assessment:** No sign. correlation between adaptive test scores and age at diagnosis, number of preceding metabolic crises, number of hospitalizations, age at transplantation
Muelly et al., 2013 *(USA)* [24]	MSUD	26	*Reviewed sample:* IT-IEM patients on diet, not liver-transplanted *Orig. sample:* IT-IEM patients on diet and IT-IEM after liver transplantation (N = 37)	• For MSUD diet n = 26: n.a., Mdn = 19.5 (7 to 35)	Healthy control group (mostly siblings of MSUD-patients)	• SCID *(adult or childhood version)* for DSM-IV: depression, anxiety, ADHD, global, social, occupational and psychological functioning *(self)*	**• Psychological adjustment (Severity of depression and Anxiety, BDI, BAI, BYI):** No sign. group difference
							**• Psychological adjustment (Current and lifetime depression and anxiety, SCID for DSM-IV):** Sign. more lifetime depression and anxiety
				• For controls n = 26: n.a., Mdn = 15.9 (6 to 35)		• BDI and BAI or sub-scores of the BYI of emotional and social impairment *(self)*	**• Risk factor assessment:** Patients who remained asymptomatic throughout newborn period vs. patients who were encephalopathic at the time of diagnosis: Second group has higher risk to later suffer from anxiety (5x higher) and from depression (10x higher)
							**• Risk factor assessment:** Correlation of mood disturbances with some biochemical parameters. No strong correlation of depression and anxiety with indices of lifetime metabolic control
Packman et al., 2007 (*USA)* [28]	MSUD	55	Same	11 (5 to 18)	Population norms	• PedsQL *(self, proxy-parent)*	**• HrQoL (PedsQL):** Total HrQoL score and domains are closer to cancer sample norms than to healthy sample norms
						• BASC *(proxy-parent, proxy-teacher)*	**• Psychological adjustment and adaptive functioning (BASC):** Mostly no sign. group difference. Sign. more problems in some areas, sign. lower scores in adaptive skills (parent- and teacher-rating)
							**• Self- vs. proxy-rating:** HrQoL self-report > proxy-report for physical, emotional, social domain, no difference for school function
							**• Self- vs. proxy-rating:** Behavioural adjustment proxy parent- vs. proxy teacher-report: parent < teacher for internalising problems (somatization, anxiety)
Pohorecka et al., 2012 *(Poland)*[29]	TYR I	8	Same	11 (6 to 15)	Population norms	• CBCL *(proxy-parent)*	**• Psychological adjustment (CBCL):** Sign. more problems in several scales
Simons et al., 2006 *(Belgium)* [21]	OTCD, GA III, MMA	11	*Reviewed sample:* only IT-IEM *Orig. sample:* IT-IEM mixed with other IEM (N = 53)	n.a. (0-2 to 16) (for N = 53)	Population norms	• CBCL, TRF, YSR *(proxy-parent, proxy-teacher, self if child > 11 years old)*	**• Psychological adjustment (CBCL):** No sign. group difference
						• K-SADS for DSM-IV diagnosis *(self)*	**• Psychological adjustment (K-SADS for DSM-IV):** Psychiatric diagnoses in n = 2, but scale was not applied to the whole sample

*The N reported corresponds to the highest number of participants for which HrQoL/psychological outcome is reported.

**Results are based on the statistic analysis done for this review. A “significant” outcome means that the calculated 95% CI of the effect size does not include the value of zero and is thus significant on a level of p <0.05 (continuous results) or that the χ^2^−test revealed a significant result on a level of p <0.05 or lower (dichotomous results). The statements refer to IT-IEM patients related to the respective group of comparison.

**Abbreviations diseases:** AMD (Amino Acid Metabolism Disorders), ASA (Arginosuccinic Aciduria), ASLD (Adenylosuccinate Lyase Deficiency), CMD (Carbohydrate Metabolism Disorders), GA I (Glutaric Aciduria type I), GA III (Glutaric Aciduria type III), HHH Syndrom (Hyperornithinemia-Hyperammonemia-Homocitrullinuria Syndrome), IEM (Inborn Errors of Metabolism), IVA (Isovaleric Aciduria), IT-IEM (Intoxication-type Inbron Errors of Metabolism), MMA (Methylmalonic Aciduria), MSUD (Maple Syrup Urine Disease), OTCD (Ornithintranscarbamylase Deficiency), PA (Propionic Aciduria), TYR I (Tyrosinemia type I), UCD (Urea Cycle Disorder).

**Abbreviations assessment instruments:** ABAS (Adaptive Behavior Assessment System), BAI (Beck Anxiety Inventory), BASC (Behavior Assessment System for Children), BDI (Beck Depression Inventory), BYI (Beck Youth Inventory), CBCL (Child Behaviour Check List) with YSF (Youth-report form) and TRF (Teacher-report form), DSM-IV (Diagnostic and Statistical Manual of Mental Disorders IV), Kiddy-, Kid-, Kiddo-KINDL (Revised questionnaire to assess health-related quality of life in children and adolescents), K-SADS (Schedule for Affective Disorders and Schizophrenia for School-Age Children), PedsQL (Pediatric Quality of Life Inventory), SDQ (Strengths and Difficulties Questionnaire), SCID (Structured clinical interview for DSM-IV), Vineland (Vineland Adaptive Behavior Scale), WHOQOL-100 (World Health Organisation Quality of Life assessment).

**Table 2:** **Overview of assessment instruments in the reviewed studies**

**Assessment instrument**		**General use**	**Reference**
**ABAS**	Adaptive behavior assessment system	Assessment of adaptive behaviour and skills necessary for daily living, for individuals from birth to 89 years. Thirteen scales are organised in three general areas: conceptual, social, practical. Versions for self- and different proxy-reports are available.	Harrison, PL, Oakland, T (2003). *Adaptive Behavior Assessment System - Second Edition.* San Antonio, TX: The Psychological Corporation.
**BAI**	Beck anxiety inventory	Assessment of severity of anxiety of individuals aged from 17 to 80 years. Consists of 21 multiple choice questions for self-report.	Beck, A, Steer, R (1993). *Manual for the Beck Anxiety Inventory.* San Antonio, Texas, USA: The Psychological Corporation Harchourt Brace & Company; 1993.
**BASC**	Behavior assessment system for children	Assessment of behaviour and self-perception of children aged from 2 years 6 months to 18 years. Teacher-, parent- and self-report versions available.	Reynolds CR, Kamphaus RW: *Behavior assessment system for children.* Circle Pines, MN: American Guidance Service 1992.
**BDI**	Beck depression inventory	Assessment of severity of depression of individuals aged from 13 to 80 years. Consists of 21 multiple choice questions for self-report.	Beck AT, Ward CH, Mendelson M, Mock J, Erbaugh J: **An inventory for measuring depression.** *Arch Gen Psychiatry* 1961, **4:**561**–**571.
**BYI**	Beck youth inventory	Consisting of five inventories (anger, anxiety, depression, disruptive behaviour, self-concept) for children and adolescents aged from 7 to 17 years. Each inventory consists of 20 questions for self-report.	Beck, J, Beck, A, Jolly, J (2001). Beck Youth Inventories of Emotional and Social Impairment. San Antonio, Texas USA: The Psychological Corporation.
**CBCL**	Child behaviour check list	Ratings of behavioural, emotional and social functioning of children and adolescents aged from 1 year 6 month to 18 years. Behaviours are categorized into internalising problem scales (e.g. anxiety, somatic complaints) and externalising problem scales (e.g. aggressive behaviour, attention problems). The CBCL is for parent-report, a teacher-report form (TRF) and a youth-report form (YRF) are available.	Achenbach, TM, & Rescorla, LA (2000). *Manual for the ASEBA Preschool Forms & Profiles.* Burlington, VT: University of Vermont, Research Center for Children, Youth, & Families.
**KINDL**	Revised questionnaire to assess health-related quality of life in children and adolescents	Generic instrument to assess health-related quality of life in children and adolescents aged from 3 to 17 years. Version for three age groups are available (Kiddy-, Kid-, Kiddo-KINDL), each in self- and proxy-rating. Dimensions: psychological well-being, social relationships, physical function, everyday life activities.	Ravens-Sieberer U, Bullinger M: **Assessing health-related quality of life in chronically ill children with the German KINDL: first psychometric and content analytical results.** *Qual Life Res* 1998, **7:**399**–**407.
**K-SADS**	Schedule for affective disorders and schizophrenia for school-age children	Semi-structured interview to make DSM-IV (Diagnostic and Statistical Manual of Mental Disorders IV) diagnoses in children and adolescents from aged from 6 to 16 years. Answers from parents and children are both considered.	Kaufman J, Birmaher B, Brent D, Rao U, Flynn C, Moreci P, Williamson D, Ryan N: **Schedule for Affective Disorders and Schizophrenia for School-Age Children-Present and Lifetime Version (K-SADS-PL): initial reliability and validity data.** *J Am Acad Child Adolesc Psychiatry* 1997, **36:**980**–**988.
**PedsQL**	Pediatric quality of life inventory	Assessment of health-related quality of life in children and adolescents aged from 2 to 18 years. Can be used in healthy individuals (generic module) and in those with health conditions (additional disease-specific modules). Self- and proxy-report versions are available. Consists of 23 items forming the generic module. Disease-specific modules are available e.g. for asthma, diabetes, cancer. Scales: Physical, emotional, social and school functioning.	Varni JW, Seid M, Rode CA: **The PedsQL: measurement model for the pediatric quality of life inventory.** *Med Care* 1999, **37:**126**–**139.
**SCID**	Structured clinical interview for DSM-IV	Semi-structured interview to make DSM-IV (Diagnostic and Statistical Manual of Mental Disorders IV) diagnoses in adults, version for children is available.	First MB, Spitzer, RL, Gibbon, M, Williams, JBW: *Structured clinical interview for DSM-IV_TR Axis I Disorders, Research Version, Non-Patient Edition.* New York, NY: Biometrics Research, New York State Psychiatric Institute; 2002.
**SDQ**	Strengths and difficulties questionnaire	Instrument to screen behavioural strengths and difficulties in children and adolescents aged from 3–16 years. Parent- or teacher-report, available in self-report for 11–16 year olds. 25 Items for 5 scales: emotional symptoms, conduct problems, hyperactivity/inattention, peer relationship problems, prosocial behaviour.	Goodman R: **The Strengths and Difficulties Questionnaire: a research note.** *J Child Psychol Psychiatry* 1997, **38:**581**–**586.
**Vineland**	Vineland adaptive behavior scale	Assessment of adaptive behaviour and skills necessary for daily living from birth to 90 years. Scales refer to functions necessary for daily living and are organised in three main areas: Communication, daily living skills, socialization. Self-, caregiver- and teacher-rating forms are available.	Sparrow SS, Cicchetti, DV, Balla, DA: *Vineland Adaptive Behavior Scales.* Circle Pines, MN: AGS Publishing; 2005.
**WHOQOL-100**	World health organisation quality of life assessment	Instrument to assess subjective quality of life in adults. Self- and proxy-report version available. Dimensions: physical, psychological, independence, social, environment, religion/spirituality.	The WHOQOL Group: **The World Health Organization Quality of Life Assessment (WHOQOL): development and general psychometric properties.** *Soc Sci Med* 1998, **46:**1569**–**1585.

**Table 3:** **Continuous outcomes in the reviewed studies**

**Reference**				**CI of d**			
**N**	**Instrument**	**Subscale**	**ES** ^**1**^ **: Cohen's d**	**Lower**		**Upper**	**Sign.**
**Health-related quality of life: self-report**							
			**Compared to population norms**				
Cazzorla et al., 2012 [22]	WHOQOL-100	General	n.a.	n.a.	to	n.a.	n.a.
N = 15		Physical	0.62	0.10	to	1.13	*
		Psychological	0.52	0.00	to	1.04	ns
		Independence	n.a.	n.a.	to	n.a.	n.a.
		Social	−0.13	−0.64	to	0.39	ns
		Environmental	−2.43	−2.97	to	−1.89	*
		Spiritual	n.a.	n.a.	to	n.a.	n.a.
		Medication	n.a.	n.a.	to	n.a.	n.a.
			**Compared to PKU**				
		General	0.03	−0.69	to	0.74	ns
		Physical	0.16	−0.56	to	0.88	ns
		Psychological	0.70	−0.04	to	1.44	ns
		Independence	−0.34	−1.06	to	0.38	ns
		Social	0.14	−0.58	to	0.85	ns
		Environmental	0.41	−0.31	to	1.14	ns
		Spiritual	0.31	−0.41	to	1.04	ns
		Medication	0.70	−0.04	to	1.44	ns
			**Compared to Morbus Fabry**				
		General	1.15	0.35	to	1.95	*
		Physical	1.49	0.65	to	2.33	*
		Psychological	0.90	0.12	to	1.68	*
		Independence	1.11	0.31	to	1.90	*
		Social	0.70	−0.06	to	1.47	ns
		Environmental	0.91	0.13	to	1.69	*
		Spiritual	1.07	0.28	to	1.87	*
		Medication	−0.35	−1.10	to	0.40	ns
			**Compared to IEM with pharmacological treatment**				
		General	0.92	0.27	to	1.56	*
		Physical	1.19	0.53	to	1.86	*
		Psychological	0.94	0.30	to	1.59	*
		Independence	0.74	0.11	to	1.38	*
		Social	0.16	−0.46	to	0.78	ns
		Environmental	0.75	0.12	to	1.39	*
		Spiritual	0.90	0.25	to	1.54	*
		Medication	−0.26	−0.88	to	0.36	ns
			**Compared to population norms**				
Grünert et al., 2013 [26]	KINDL	Total	−0.34	−0.81	to	0.12	ns
N = 18		Physical	−0.28	−0.74	to	0.19	ns
		Psychological	−0.78	−1.25	to	−0.32	*
		Self-esteem	0.15	−0.32	to	0.63	ns
		Family	−0.38	−0.84	to	0.09	ns
		Friends	−0.68	−1.14	to	−0.21	*
		School	0.73	0.22	to	1.24	*
		Illness	n.a.	n.a.	to	n.a.	n.a.
			**Compared to healthy population norms**				
Packman et al., 2007 [28]	PedsQL self-report	Physical function	−0.28	−0.69	to	0.13	ns
N = 55		Emotional function	−0.55	−0.96	to	−0.14	*
		Social function	−0.80	−1.22	to	−0.39	*
		School function	−0.70	−1.12	to	−0.29	*
		Psychosocial	−0.85	−1.27	to	−0.44	*
		Total	−0.77	−1.19	to	−0.36	*
			**Compared to cancer population norms**				
		Physical function	0.42	0.00	to	0.84	ns
		Emotional function	−0.04	−0.46	to	0.38	ns
		Social function	−0.24	−0.66	to	0.19	ns
		School function	−0.24	−0.67	to	0.18	ns
		Psychosocial	−0.23	−0.65	to	0.19	ns
		Total	−0.02	−0.44	to	0.40	ns
**Health-related quality of life: proxy-report**							
			**Compared to CMD**				
Eminoglu et al., 2013 [23]	QOL scale for metabolic disease	Impact of IEM	−0.18	−1.02	to	0.67	ns
N = 14		Attention	−0.42	−1.15	to	0.31	ns
		Self-esteem about IEM	0.03	−0.70	to	0.76	ns
		Physical function	−0.65	−1.38	to	0.08	ns
		Labeling	−0.57	−1.41	to	0.27	ns
		Social support	0.17	−0.69	to	1.03	ns
		School status	−0.93	−1.77	to	−0.09	*
		Health perception	−1.69	−2.64	to	−0.74	*
	KINDL	Emotional wellbeing	−0.44	−1.07	to	0.19	ns
			**Compared to AMD**				
	QOL scale for metabolic disease	Impact of IEM	−0.24	−1.18	to	0.69	ns
		Attention	−0.64	−1.43	to	0.16	ns
		Self-esteem about IEM	−0.45	−1.26	to	0.36	ns
		Physical function	−1.14	−1.96	to	−0.33	*
		Labeling	−0.15	−1.02	to	0.71	ns
		Social support	−0.55	−1.47	to	0.38	ns
		School status	−1.41	−2.41	to	−0.40	*
		Health perception	−1.67	−2.70	to	−0.64	*
	KINDL	Emotional wellbeing	−0.20	−0.88	to	0.48	ns
			**Compared to population norms**				
	QOL scale for metabolic disease		n.a.	n.a.	to	n.a.	n.a.
	KINDL		n.a.	n.a.	to	n.a.	n.a.
			**Compared to healthy population norms**				
Packman et al., 2007 [28]	PedsQL proxy-report	Physical function	−1.14	−1.54	to	−0.74	*
N = 55		Emotional function	−1.19	−1.59	to	−0.80	*
		Social function	−1.48	−1.88	to	−1.08	*
		School function	−1.46	−1.86	to	−1.06	*
		Psychosocial	−1.66	−2.06	to	−1.26	*
		Total	−1.65	−2.05	to	−1.25	*
			**Compared to cancer population norms**				
		Physical function	−0.06	−0.46	to	0.34	ns
		Emotional function	−0.23	−0.63	to	0.17	ns
		Social function	−0.65	−1.05	to	−0.25	*
		School function	−0.39	−0.80	to	0.01	ns
		Psychosocial	−0.54	−0.94	to	−0.14	*
		Total	−0.40	−0.80	to	0.00	ns
**Psychological adjustment and adaptive functioning: self-report**							
			**Compared to population norms**				
Muelly et al., 2013 [24]	BDI/BYI	BDI score adults	0.54	−0.02	to	1.11	ns
N = 26		BYI T-score	−0.44	−1.01	to	0.12	ns
		Combined z-score	0.19	−0.36	to	0.75	ns
	BAI/BYI	BAI score adults	0.38	−0.18	to	0.94	ns
		BYI T-score	−0.28	−0.83	to	0.28	ns
		Combined z-score	0.13	−0.43	to	0.68	ns
			**Compared to population norms**				
Mazariegos et al., 2012 [20]	Vineland or ABAS	Total	−1.09	−1.46	to	−0.72	*
N = 31							
**Psychological adjustment and adaptive functioning: proxy-report**							
			**Compared to population norms**				
Beauchamp et al., 2009 [25]	CBCL	Internalising problems	−0.32	−1.31	to	0.66	ns
N = 4		Externalising problems	−0.76	−1.74	to	0.23	ns
		Total	−1.32	−2.31	to	−0.34	*
	ABAS	General score	−0.23	−1.21	to	0.75	ns
		Conceptual	−0.65	−1.63	to	0.34	ns
		Social	0.84	−0.14	to	1.82	ns
		Practical	−0.50	−1.48	to	0.48	ns
			**Neonatal onset vs. population norms**				
Krivitzky et al., 2009 [27]	ABAS, age <3 y	General score	−0.35	−0.83	to	0.13	ns
N = 92	ABAS, age 3-16y	General score	−2.09	−2.64	to	−1.53	*
			**Late onset vs. population norms**				
	ABAS, age <3 y	General score	−0.67	−1.42	to	0.07	ns
	ABAS, age 3-16y	General score	−0.76	−1.04	to	−0.47	*
			**Hyp.amm. events > 0 vs. population norms**				
	ABAS, age <3 y	General score	−0.37	−0.88	to	−0.37	*
	ABAS, age 3-16y	General score	−1.00	−1.33	to	−0.99	*
			**Hyp.amm. events =0 vs. population norms**				
	ABAS, age <3 y	General score	−0.55	−1.20	to	0.11	ns
	ABAS, age 3-16y	General score	−0.96	−1.36	to	−0.56	*
			**Compared to population norms**				
Packman et al., 2007 [28]	BASC parent-report	Hyperactivity	0.26	−0.03	to	0.55	ns
N = 55		Aggression	0.24	−0.04	to	0.53	ns
		Conduct problems	0.03	−0.25	to	0.32	ns
		Anxiety	0.05	−0.24	to	0.34	ns
		Depression	0.08	−0.21	to	0.36	ns
		Somatization	0.21	−0.08	to	0.50	ns
		Atypicality	0.30	0.01	to	0.59	*
		Withdrawal	0.04	−0.24	to	0.33	ns
		Attention problems	0.68	0.39	to	0.97	*
		Externalising problems	0.21	−0.08	to	0.50	ns
		Internalising problems	0.14	−0.15	to	0.43	ns
		Behavioral sympt. index	0.37	0.08	to	0.66	*
		Adaptive skills	−0.59	−0.88	to	−0.30	*
			**Compared to population norms**				
	BASC teacher-report	Hyperactivity	0.41	0.07	to	0.76	*
		Aggression	0.28	−0.07	to	0.62	ns
		Conduct problems	−0.18	−0.52	to	0.16	ns
		Anxiety	0.55	0.20	to	0.89	*
		Depression	0.11	−0.23	to	0.45	ns
		Somatization	0.66	0.32	to	1.01	*
		Attention problems	0.75	0.41	to	1.10	*
		Learning problems	0.74	0.39	to	1.08	*
		Atypicality	0.68	0.34	to	1.03	*
		Withdrawal	0.19	−0.15	to	0.53	ns
		Externalising problems	0.23	−0.12	to	0.57	ns
		Internalising problems	0.56	0.21	to	0.90	*
		School problems	0.76	0.42	to	1.11	*
		Behavioral sympt. index	0.62	0.28	to	0.97	*
		Adaptive Skills	−0.36	−0.71	to	−0.02	*
			**Compared to population norms**				
Pohorecka et al., 2012 [29]	CBCL	Internalising problems	1.14	0.44	to	1.84	*
N = 8		Externalising problems	0.77	0.08	to	1.47	*
		Withdrawn	1.30	0.60	to	2.01	*
		Somatic complaints	1.19	0.49	to	1.90	*
		Anxious depressed	0.62	−0.08	to	1.32	ns
		Social problems	1.52	0.82	to	2.23	*
		Thought problems	0.89	0.19	to	1.59	*
		Attention problems	1.15	0.45	to	1.86	*
		Rule breaking behaviour	1.07	0.37	to	1.77	*
		Aggressive behaviour	0.86	0.16	to	1.56	*
			**Compared to population norms**				
Simons et al., 2006 [21]	CBCL	Internalising problems	0.79	−0.02	to	1.59	ns
N = 11		Externalising problems	0.36	−0.44	to	1.17	ns
		Total	0.64	−0.17	to	1.44	ns

^1^A positive value means that the IT-IEM group scored higher than the control group. A negative value means that the IT-IEM group scored lower than the control group. Higher values are favourable in all scales except for the BASC [28] where high scores mean more problems (exception: BASC subscale adaptive skills).

**Abbreviations:** ABAS (Adaptive Behavior Assessment System), BAI (Beck Anxiety Inventory), BASC (Behavior Assessment System for Children), BDI (Beck Depression Inventory), BYI (Beck Youth Inventory), CBCL (Child Behaviour Check List), KINDL (Revised questionnaire to assess health-related quality of life in children and adolescents), PedsQL (Pediatric Quality of Life Inventory), Vineland (Vineland Adaptive Behavior Scale), WHOQOL-100 (World Health Organization Quality of Life assessment).

**Table 4:** **Dichotomous outcomes in the reviewed studies**

**Reference N**	**Instrument**	**Subscale**	**n IT-IEM**	**n control**	**Results**	**Test of between group significance**	**Cramer's V**
**Psychological adjustment, self-report**							
					**% present IT-IEM/% present control**	**Compared to healthy control group**	
Muelly et al., 2013 [24]	SCID, DSM-IV	Depression current	26	26	29/4	χ^2^ = 22.68; *df* = 1; *p* (2-tail) = 0.000 (***)	0.34
N = 26		Depression lifetime	26	26	42/19	χ^2^ = 12.48; *df* = 1; *p* (2-tail) = 0.000 (***)	0.25
		Anxiety current	26	26	42/15	χ^2^ = 17.89; *df* = 1; *p* (2-tail) = 0.000 (***)	0.30
		Anxiety lifetime	26	26	58/31	χ^2^ = 14.96; *df* = 1; *p* (2-tail) = 0.000 (***)	0.27
**Psychological adjustment, proxy-report**							
					**n IT-IEM in different categories (n control n.a.)**	**No group of comparison**	
Gramer et al., 2013 [19]		Perceived burden for the child	34	none	No (n = 5), little (n = 17), middle (n = 5), heavy (n = 4), very heavy (n = 3)	n.a.	n.a.
N = 34							
		Social behaviour	34	none	Lower than norm (n = 3), same as norm (n = 28), higher than norm (n = 3)	n.a.	n.a.
					**% normal/at risk/clinically sign. IT-IEM**	**Compared to population norms**	
					**(% normal/at risk/clinically sign. control)**		
Grünert et al., 2013 [26]	SDQ	Emotional symptoms	48	930	62/13/25 (84/7/9)	χ^2^ = 12.64; *df* = 2; *p* (2-tail) = 0.002 (**)	0.25
N = 48		Conduct problems	46	930	54/26/20 (69/16/15)	χ^2^ = 4.93; *df* = 2; *p* (2-tail) = 0.085 (ns)	0.15
		Hyperactivity/Inattention	47	930	62/28/10 (86/6/8)	χ^2^ = 18.35; *df* = 2; *p* (2-tail) = 0.000 (***)	0.30
		Peer relationship problem	46	930	39/20/41 (78/11/12)	χ^2^ = 32.92; *df* = 2; *p* (2-tail) = 0.000 (***)	0.40
		Prosocial behaviour	47	930	57/9/34 (89/7/4)	χ^2^ = 30.95; *df* = 2; *p* (2-tail) = 0.000 (***)	0.39
		Influence on child's life	48	930	60/11/29 (n.a.)	n.a.	n.a.
		Total	46	930	52/20/28 (85/8/7)	χ^2^ = 25.69; *df* = 2; *p* (2-tail) = 0.000 (***)	0.36
					**% normal/at risk/clinically sign. IT-IEM**	**Compared to population norms**	
					**(% normal/at risk/clinically sign. control)**		
Krivitzky et al., 2009 [27]	CBCL	Internalising problems	68	576	79/17/4 (83/7/11)	χ^2^ = 5.87; *df* = 2; *p* (2-tail) = 0.053 (ns)	0.17
N = 92		Externalising problems	68	576	80/16/4 (83/7/11)	χ^2^ = 5.26; *df* = 2; *p* (2-tail) = 0.072 (ns)	0.16

**Abbreviations:** CBCL (Child Behaviour Check List), DSM-IV (Diagnostic and Statistical Manual of Mental Disorders IV), SCID (Structured Clinical Interview for DSM-IV), SDQ (Strengths and Difficulties Questionnaire).

Many of the studies had methodological limitations. One main weakness is the lack of appropriate participant selection (i.e. use of convenience samples) in order to avoid selection or non-response bias [22–29]. Two studies involved a non-validated measuring tool [19,23]. Furthermore, only two studies considered multiple informants in terms of self- and proxy-ratings [21,28].

### Findings on HrQoL

#### Self-report

As shown in Table 3, results for self-reported HrQoL are inconsistent across studies. In one study, the authors reported significantly lower HrQoL for IT-IEM patients in most domains compared to population norms [28]. The results for IT-IEM patients were closer to the results of a cancer population than to a healthy one. Two other studies showed results with more variance throughout the different HrQoL domains. One study reported clearly lower scores for IT-IEM patients than for population norms in the *psychological* and the *friends domain*s, but higher scores for the *school domain* [26]. The results for other domains did not differ from norms. Half of the patients in the sample of this study did not categorise themselves as “ill”. In contrast, another group of researches reported significantly better scores in the *physical domain* for IT-IEM patients compared to population norms [22], but lower scores in the *environmental domain* (including e.g. financial resources, health care, physical environment). The same study found HrQoL of IT-IEM patients to be similar to that of patients with phenylketonuria, who also have to follow a strict diet but who do not face the risk of sudden metabolic crises. Furthermore, IT-IEM patients had higher HrQoL in most domains compared to Morbus Fabry patients, who often have to deal with pain symptoms, and patients with metabolic diseases under pharmacological (as opposed to dietary) treatment [22].

#### Proxy-report

Proxy-ratings of HrQoL were all reported by parents. All results for IT-IEM patients were either not different from norms or unfavourable for IT-IEM patients (Table 3). One study showed significantly lower parent-rated HrQoL for IT-IEM patients across all domains compared to population norms [28]. In line with the results for self-reported HrQoL in the same study, the results were closer to cancer population norms than to healthy norms. *Psychosocial* and *social domains* were lower in IT-IEM patients than in the cancer population. In another article, the parent-rated HrQoL of IT-IEM patients was compared with the parent-rated HrQoL of patients with other metabolic diseases (aminoacid metabolic disorders and carbohydrate metabolic disorders) [23]. IT-IEM patients scored lower in the domains of *school functioning*, *health perception* (compared with both diseases) and *physical functioning* (only compared with aminoacid metabolic disorders).

#### Self- vs. proxy-report

One study [28] compared self- and parent proxy-ratings. The authors reported significantly better scores in self-reported HrQoL for the domains of *physical*, *emotional* and *social* HrQoL, but no difference for *school functioning*.

#### Risk factors

No risk factors regarding HrQoL were investigated in the studies.

### Findings on psychological adjustment and adaptive functioning

#### Self-report

Results for self-reported psychological adjustment and adaptive functioning are shown in Tables 3 and 4. Depression and anxiety (both current and lifetime) were reported to be more prevalent in a group of IT-IEM patients than in a healthy group [24]. However, the study revealed no differences between IT-IEM patients and population norms regarding anxiety and depression symptom severity. Another research group found adaptive functioning for IT-IEM patients to be significantly lower than in a norm population [20].

#### Proxy-report

All studies used parent proxy-reports and one additionally used teacher proxy-reports (Tables 3 and 4). Proxy-report findings on different aspects of psychological adjustment and adaptive functioning mostly showed either no difference or worse adjustment and more problems for IT-IEM patients than norms [21,26–29]. Fewer problems compared to population norms were described in one paper [25].

#### Proxy-parent vs. proxy-teacher-report

According to one study, teachers reported more *internalising problems* (somatisation, anxiety) than parents [28].

#### Risk factors

For psychological adjustment and adaptive functioning parameters, several risk factors were investigated. The risk for *lifetime anxiety* or *lifetime depression disorders* was higher in patients who were encephalopathic at diagnosis than patients who were non-symptomatic at diagnosis [24]. Adaptive functioning correlated with IQ according to another study, but no correlation was found between adaptive functioning and age at diagnosis, number of preceding metabolic crises, or number of hospitalisations [20]. Finally, lower scores in adaptive functioning were found among patients with neonatal onset than among late-onset patients, but no difference was found in the number of hyperammonemic events [27].

## Discussion

### HrQoL, psychological adjustment, and adaptive functioning

The first aim of our systematic review was to explore the current state of research on HrQoL, psychological adjustment, and adaptive functioning in patients with IT-IEM. We found 11 articles reporting HrQoL, psychological adjustment, or adaptive functioning for this group of patients. Results for HrQoL varied across studies from lower HrQoL to similar and better scores for IT-IEM patients compared to norms. Notably, proxy-ratings of patients’ HrQoL were consistently similar or lower than norms. Results for psychological adjustment and adaptive functioning varied less and were mostly comparable to norms or showed worse adjustment for IT-IEM patients. Fewer problems were only reported once [25] – however, this result has to be considered with care. The sample size of this study was very small (n = 4) compared to the other studies, and the authors themselves expressed some doubts about the reliability of their results.

Impaired HrQoL, psychological adjustment, or adaptive functioning can have different causes. Firstly, this may be a result of the distress experienced in IT-IEM, such as fear of metabolic crises or social problems associated with the diet [9]. Secondly, neurological sequelae of IT-IEM can lead directly to cognitive or psychological problems and thus to worse psychological adjustment and adaptive functioning in everyday life (e.g. psychotic or depressive symptoms through CNS damage) [5]. Thirdly, it is important to consider the interaction between HrQoL and psychological adjustment, since the literature shows impaired HrQoL in patients with mental disorders [7]. Therefore, impaired HrQoL may be caused by mental health problems. In contrast, good HrQoL in IT-IEM patients may be explained by the theory of response shift [30]. This would account for the often-seen improvement of HrQoL in chronically ill patients as a result of an accommodation process which involves changing internal standards, values and conceptualisation. According to Sprangers and Schwartz [30], a response shift results from the interaction of different variables: health status, mechanisms such as coping, and antecedents such as personality or sociodemographics.

Interestingly, HrQoL was more often reported to be impaired when rated by parents compared to self-ratings by patients. In line with this, a comparison of self- and proxy-ratings revealed better self-ratings in most domains [28]. The fact that HrQoL of children with chronic health conditions is rated lower by parents than by the children themselves is well known from the literature [31]. Furthermore, there is a close relation between the parent’s rating of a child’s HrQoL and the parent’s own HrQoL [32]: Parents experiencing low HrQoL rate their child’s HrQoL low as well. This might be especially relevant in parents of children with IT-IEM, since these diseases demand intensive care and may have a great impact on the lives of caregivers [33]. Another explanation for the lower proxy-ratings may be the ability of parents to anticipate the future problems of the child. Young children in particular may not be aware of these to the same extent.

A major reason for the inconsistent findings may be attributed to methodological issues. Most of the HrQoL instruments were not specifically tailored to patients with IT-IEM. One study used an invalidated disease-specific instrument [23]. In addition, the HrQoL of IT-IEM patients was compared to different groups: population norms or other metabolic diseases. Sample sizes were often small, a state of affairs that is often found in paediatric research and especially in research in the field of rare diseases. Statistically, such small sample sizes make it more difficult to detect group differences. In addition, one study had a clear selection bias by only including patients who were cognitively able to answer questionnaires [26]. Another significant limitation is the choice of informants; most studies used proxy-reports which do not fully reflect self-reports of the patients. It is clear from the literature that these two kinds of reports are not interchangeable [32].

### Risk factors for HrQoL, psychological adjustment, and adaptive functioning

The second aim of this review was to detect risk factors for HrQoL, psychological adjustment, and adaptive functioning for IT-IEM patients. Only a few risk factors for psychological adjustment and adaptive functioning have been investigated so far, and none have been examined for HrQoL. The risk factors examined were mainly medical [20,24,27]. With regard to cognitive parameters, IQ was investigated in one study [20]. Two studies revealed that metabolic events in the neonatal period were associated with psychosocial adjustment: patients diagnosed as encephalopathic newborns had a higher risk of suffering from anxiety or depression during their lifetime than patients who were metabolically stable during the newborn period [24]. Adaptive functioning scores were lower for neonatal-onset than for late-onset patients [27]. Similar results have been found before and may be explained by the fact that crises have a negative impact on the developing brain, especially in highly vulnerable newborns [24,34].

### Strengths and limitations of this review

This systematic review was conducted in accordance to the PRISMA guidelines (Preferred Reporting Items for Systematic Reviews and Meta-Analysis) [35]. In order to find as many eligible studies as possible, we applied several search strategies: we searched different databases and did not restrict the search to English articles. Additionally, we tried to find additional and/or unpublished studies by reference screening and contacting experts in the field. Study selection and data extraction were conducted independently by two reviewers, thereby diminishing the risk of bias. The main weakness of our review is the inability to pool results from the different studies. The different study designs and assessment methods did not permit such meta-analytic calculations. Nevertheless, better comparability of the results was attained by indicating effect sizes and their CI. Another limitation of this systematic review can be found in our comparison of studies with very different sample sizes (ranging from N = 4 to N = 92), the reason why the calculated effect sizes have to be interpreted carefully. Because the incidence rates of rare diseases are small and because there is only a very limited number of studies that could be included in our review, we decided not to exclude studies with small sample sizes (inclusion criterion N >1). To account for this limitation, we considered sample sizes in the calculation of effect sizes by correcting mechanisms. The different sizes were also taken into account by computing CIs of effect sizes.

### Suggestions for future research

Based on the findings of this review, several implications can be drawn. Firstly, HrQoL should be considered as an essential outcome parameter in future clinical trials. Up to now, treatment evaluations of patients with IT-IEM have predominantly focused on medical outcomes. However, because survival rates have increased considerably, improvement of HrQoL for patients with IT-IEM must be an additional major goal of new treatments.

Secondly, it is important to increase the methodological quality of psychosocial research among IT-IEM patients. Multicentre studies are necessary both to avoid convenience sampling with a high risk for biased data and to increase sample sizes. International patient registries (e.g. E-IMD, www.e-imd.com) can help to achieve this goal and to aggregate knowledge. This review has shown that a variety of assessment instruments is currently in use, thus complicating the pooling of results. Most of the studies used generic, non-disease-specific instruments. Only one of the reviewed studies used a disease-specific scale [23]; however, this was not validated. Generic instruments have the advantage that results can usually be compared to a healthy population or a population with another disease [6]. However, they do not assess the specific problems of medical conditions. In contrast, disease-specific HrQoL scales are related to the distinct effects of a particular disease [5]. They are more sensitive for special topics that are of interest and more meaningful for specific groups of patients. The use of disease-specific HrQoL assessment measures in clinical trials has been shown to be valuable in other severe medical conditions [5]. To date, no validated, disease-specific measure is available for patients with IT-IEM. Furthermore, from a methodological viewpoint, it is desirable to consider different informants; because proxy-reports have several limitations, the patients’ well-being should also be assessed using self-ratings.

Thirdly, knowledge of risk factors influencing HrQoL, psychological adjustment, and adaptive functioning is of great importance. Very few studies have addressed this topic at all, and where they did, research was restricted to medical or biochemical parameters or IQ. Medical parameters are weak predictors for HrQoL in most chronic diseases [5]. In contrast, a systematic review exploring HrQoL in rare genetic conditions has emphasised that parameters explaining how patients coped with their disease were good predictors of HrQoL [5]. As an example, the authors mentioned scales such as “acceptance of disability” or “sense of coherence”, which were positive predictors for QoL, while feeling hopeless or having a fatalistic view correlated with lower QoL [5]. Other predictors for HrQoL in chronic disease described in the literature include concepts such as locus of control, attachment, or well-being of the parents [8,36,37]. In IT-IEM patients, such individual and familial psychosocial risk factors have not yet been studied. Current research is, unfortunately, still focused on a fully biomedical model, instead of including the promising psychosocial perspective [5].

### Clinical implications

The small number of studies, partially inconsistent results, and the few risk factors addressed make it difficult to draw implications for clinical practice. Since HrQoL, psychological adjustment, and adaptive functioning seem to be impaired in some patients, we suggest offering psychological support for patients and their families to help them cope more effectively with the disease. Knowing more about risk factors would allow the development of targeted interventions for certain groups of patients. Overall, we consider it important to support patients and their families with a comprehensive care model, including psychological and social interventions to complement the medical ones [12,21].

## Conclusion

Research data on psychosocial factors in IT-IEM patients are generally sparse. However, the growing interest in the topic is underlined by the fact that seven of the 11 articles reviewed were published in 2012 or 2013. Further research and improved methodological quality of studies are required. Multicentre studies and the use of a standardised, disease-specific assessment tools are needed to establish HrQoL as an important additional outcome parameter in patient-centred research and clinical trials.

## Abbreviations

BASC: Behavior Assessment System for Children
CBCL: Child Behaviour Checklist
CNS: Central nervous system
HrQoL: Health-related quality of life
IEM: Inborn errors of metabolism
IT-IEM: Intoxication-type inborn errors of metabolism
MSUD: Maple syrup urine disease
OA: Organic acidurias
TYR 1: Tyrosinemia type 1
UCD: Urea cycle disorders
